# 122 Itchy Burns: Debilitating Effects of Pruritus on Daily Activity in Burn Follow-Up Care

**DOI:** 10.1093/jbcr/irae036.121

**Published:** 2024-04-17

**Authors:** Elizabeth A Shrader, Esther A Rathjen, Alexander C Eischeid

**Affiliations:** CHI Health St. Elizabeth, Lincoln, Nebraska; Nebraska Wesleyan University, Valley, Nebraska; CHI Health St. Elizabeth, Lincoln, Nebraska; Nebraska Wesleyan University, Valley, Nebraska; CHI Health St. Elizabeth, Lincoln, Nebraska; Nebraska Wesleyan University, Valley, Nebraska

## Abstract

**Introduction:**

Current practices in the long term treatment of burn wounds often ignore or only minimally respond to prolonged pruritus, or itching. In truth, the presence of pruritus in patients receiving follow-up care which negatively impacts patient lifestyles is likely under documented and not effectively treated. Quantifying and treating pruritus in a similar fashion to pain management is thus the next logical progression of burn care in an outpatient setting. In this study, we hypothesize that the presence of pruritus positively correlates to daily activity disability and warrants investigation into further intervention.

**Methods:**

All patients presenting for an outpatient appointment for treatment of burn injuries at a regional burn center were provided a 5-D Pruritus Scale questionnaire at the time of their arrival with other clinic paperwork prior to being seen by a provider. Interpreter services for non-English speaking patients and writing assistance for patients with hand injuries were provided, when necessary. The survey collected patient ratings on pruritus duration, degree, direction, disability, and distribution. Responses were de-identified and recorded in numerical form. Of the 127 submitted surveys, blank responses (n=13) and responses which struck/altered the word “itch” and replaced it with “pain” (n=2) were omitted.

**Results:**

Surveys were collected for a 6 week period with a total of 112 valid responses obtained. Of the valid surveys, 44 (39%) reported moderate (level 3/5) itching or higher with 47 patients (41%) indicating at least occasional sleep disruption/challenges resulting from pruritus. Under daily activity disability categories (leisure/social, housework/errands, and work/school), 44 (39%) reported at least occasional inability to complete at least one of the activities with 39 (35%) and 29 (26%) indicating at least occasional disability in two or three of the activities, respectively. It was determined patients reporting moderate pruritus were significantly more likely (p < 0.001) than patients only reporting minimal or mild pruritus to report daily activity disabilities (61% versus 25%, respectively) and sleep disruptions (68% versus 25%, respectively).

**Conclusions:**

This study revealed a higher than expected proportion of patients reporting pruritus leading to daily activity disability during follow-up care for burn injuries. Further, there exists a correlation of patients indicating moderate or worse pruritus with sleep and activity disruptions.

**Applicability of Research to Practice:**

The relationship between increased rates of daily activity disability and degree of pruritus indicates providers should be vigilant in screening for pruritus-related disability and supports trialing additional itch management such as oral and topical medications.

